# Fighting Colds

**Published:** 1918-03

**Authors:** E. R. Petskey


### FIGHTING COLDS

BY DR. E. R. PETSKEY

This is the time of the year when colds are prevalent everywhere.

A cold may be confined to the nasal or pulmonary (lung) portion of the respiratory tract, or to both at one and the same time. The former may be called a simple cold and the latter a compound cold, though the one may be as dangerous and severe as the other. Here we will confine our attention to colds in general.

Generally speaking, about eight people out of ten, after having contracted a cold, attribute it to the weather. That we have no excuse for throwing the blame on the weather has been proven time and time again.

It was my luck to be in a position to experiment for one year on the causes of colds. Before this time I had a cold two or three times a year, notwithstanding the fact that I had a very strong constitution, and had I not been possessed of such I would probably now be afflicted by a chronic catarrh, like hundreds of others are, who through their weakened condition are unable to throw it off. During my experiment I changed my entire mode of living in every respect. I am now fully convinced of the fact that the following are the main causes of the catching of colds:

(1) Improper food; (2) improper clothing; (3) want of exercise; (4) an insufficient supply of fresh air; (5) imagination.

### (a) IMPROPER FOOD

People who travel have a hard time getting the right kind of food, especially when they have to eat on a diner, then in a hotel, or perhaps strike a small town and fall back on a cheap restaurant, satisfying themselves with merely a piece of pie and a cup of coffee—which is not even coffee, but some cheap compound.

Improper food will bring about a weakened condition in general of the entire system, manifesting itself wherever the vitality is at its lowest.

### (b) IMPROPER CLOTHING

As soon as the mercury column begins to drop, on goes a suit of heavy woolen underwear, and in many instances even two. By using so much clothing you shut off the air supply to the body, and in turn the system is weakened. Fresh air must be allowed to circulate around the skin. Put on a heavier overcoat, but do not wear a lot of tight-fitting woolen garments. Think of the amount of perspiration these garments will absorb and how unclean and unhealthy it is to have thick woolen garments on for days that are saturated with impurities. Perspiration is an impurity excreted through the pores of the skin. Some people even sleep in these heavy garments, not even airing them at night.

### (c) WANT OF EXERCISE

When you have a cold your system is full of impurities; your purifying systems must be brought into play, and these are mainly the blood and the air you breathe into your lungs. Exercise to stir up your circulation, to force deep breathing. This will bring about not only a local, but also a general action. That is, your cold tells a good tale, it tells you that there are impurities, poisons in your system in general. These are showing themselves where the vitality is at its lowest, be it in the shape of a boil or a cold, it matters not what nature the disorder be.

### (d) INSUFFICIENT SUPPLY OF FRESH AIR

People suffering with a cold will go around the room searching for cracks to be stuffed up with paper and felt, lest a little fresh, cool air may chance to find its way in. Foolishness, ignorance—throw it off. Fresh air will not and never can give you a cold. This is just the time when your system is crying for fresh air. Exercise and breathe deeply. Expand your chest, which will separate the walls of the lungs farther away from each other and also the walls of the bronchials, the small tubular vessels carrying the air to the lungs, thereby allowing a greater volume of oxygen to enter. The body needs food in the usual sense of the word— that is, food taken by the mouth, but also food in the form of fresh air, which is by far the most important food, especially when a cold is present. One day while traveling in a street car a woman entered with what appeared to be a medium sized bundle of white linen. A few minutes later she lifted up one flap and then another, and I saw some baby veiling. This began to arouse my curiosity. Finally four layers of this veiling were raised, and to my surprise I saw a little baby face. My goodness! you poor little child—you the victim of an inexperienced, ignorant mother.

The mother did not leave the little one's face uncovered, now that she was inside a warm car; no, she kissed the dimpled cheeks and carefully placed each piece of covering back the way it was before. How this little baby ever managed to breathe remains a puzzle. Then the parent wonders why her baby is constantly sick and delicate and does not thrive the way it should. Sickness is the outcome of ignorance.

(e) IMAGINATION

You wonder what this has to do with the catching of a cold. Well, let me tell you that it has a very great deal to do with it. The mind rules the body, and not the body the mind. Many people believe the body is supreme.

You will often hear a person say, "I am afraid I am going to be sick and I am going to catch cold! Sure enough, they get some kind of an ailment. Never give in, but resist every attack a sickness makes on you. You have got the will-power to resist sickness, and if you haven't, then the quicker you cultivate it the better. Why can physicians and nurses visit the rooms of fever patients? Simply because they have no fear of the disease. They know that their mind is supreme and use it as such. Further, they are careful with their system. They realize the importance of a healthy body.

You are caught—you have a cold! What are you going to do? Use a little menthol salve, alum nasal douche? Yes, that will remove the cold, but it will not cure the cold. If you suffer with a cold, be thankful that it is only a cold. It comes to you as a warning that your system is out of order and requires cleansing. If you use some so-called cold cure you are simply contracting the mucous membranes and the impurities are forced back into the system and will stay there till a sufficient quantity has accumulated to satisfactorily battle against all resistance and break out somewhere else in another form.

Help the cold, help nature to right itself. The following treatments can easily be taken at home. If a cabinet bath be handy, take a good sweat for fifteen to twenty-five minutes—only so long as can be comfortably borne. Place a towel wrung out of cold water about the forehead and around the neck. As soon as the towel gets warm, replace by another. Drink cool water if thirsty or weak. After this take a good warm shower bath and soap the entire body; then rub well with bathbrush to cleanse the pores of the skin and to remove the excreted perspiration. Then wash off all the soap and gradually lower the temperature to cool; then dry by brisk rubbing with rough bath towel to ensure of a good and rapid reaction. As soon as dry, lie down with a sheet and light blanket covering in medium temperature room. If there be no shower bath in the house take a tub-bath instead and go through the same process. Where there is no steam cabinet, place a cold wet sheet, well wrung out, around the patient, put him to bed and place one hot-water bag to the feet and one at each side. Use plenty of warm blankets. This will soon bring on a good sweat. Another very good treatment is to apply packs to the trunk of the body. The method of applying these packs and compresses will be found in any up-to-date work on the "Rational Treatment of Disease." If you are subject to cold feet, do not toast them in front of the open fire or the radiator, but restore warmth by restoring a natural circulation in the lower extremities either through exercise, such as rising on the tip-toes or by alternating foot-baths. First place the feet in a pail of hot water, as hot as you can bear it, for about two minutes, then into cold water for from a half to one minute, and then back into the hot. Keep the water hot all the time by adding more from time to time; make, say, from four to six changes. Dry and take a few exercises.

Throw away your whisky, and quinine, metholated salve and alum water. Don't suppress the cold, but help it to rid your system of the poisons by simple natural means.—*Health Culture.*

